# Take it or leave it: prefrontal control in recreational cocaine users

**DOI:** 10.1038/tp.2015.80

**Published:** 2015-06-16

**Authors:** S Morein-Zamir, P Simon Jones, E T Bullmore, T W Robbins, K D Ersche

**Affiliations:** 1 Behavioural and Clinical Neuroscience Institute, Department of Psychology, University of Cambridge, Cambridge, UK; 2 Department of Psychology, University of Cambridge, Cambridge, UK; 3 Department of Psychology, Anglia Ruskin University, Cambridge, UK; 4 Department of Psychiatry, University of Cambridge, Cambridge, UK; 5 Cambridgeshire and Peterborough NHS Foundation Trust, Cambridge, UK; 6 Clinical Unit Cambridge, GlaxoSmithKline, Addenbrooke's Centre for Clinical Investigations, Cambridge, UK

## Abstract

Though stimulant drugs such as cocaine are considered highly addictive, some individuals report recreational use over long periods without developing dependence. Difficulties in response inhibition have been hypothesized to contribute to dependence, but previous studies investigating response inhibition in recreational cocaine users have reported conflicting results. Performance on a stop-signal task was examined in 24 recreational cocaine users and 32 healthy non-drug using control participants matched for age, gender and verbal intelligence during functional magnetic resonance imaging scanning. The two groups were further matched on traumatic childhood histories and the absence of family histories of addiction. Results revealed that recreational cocaine users did not significantly differ from controls on any index of task performance, including response execution and stop-signal reaction time, with the latter averaging 198 ms in both groups. Functional magnetic resonance imaging analyses indicated that, compared with controls, stopping in the recreational users was associated with increased activation in the pre-supplementary motor area but not the right inferior frontal cortex. Thus, findings imply intact response inhibition abilities in recreational cocaine users, though the distinct pattern of accompanying activation suggests increased recruitment of brain areas implicated in response inhibition. This increased recruitment could be attributed to compensatory mechanisms that enable preserved cognitive control in this group, possibly relating to their hypothetical resilience to stimulant drug dependence. Such overactivation, alternatively, may be attributable to prolonged cocaine use leading to neuroplastic adaptations.

## Introduction

Use and abuse of cocaine is considered a major public health issue with prevalence estimates ranging from 14 to 21 million globally.^1^ Hallmarks of cocaine dependence include lack of control underlying the compulsive persistence in drug-taking with larger amounts consumed for longer than intended, despite adverse personal and social consequences.^2^ Cognitive control involves elements of both self- and emotion-regulation, as well as top-down intentional suppression of maladaptive actions and responses. Poor cognitive control likely contributes to multiple aspects of drug abuse, including initial usage and escalation to dependence, and subsequent maintenance.^3^ Stimulant-dependent individuals exhibit behavioral impairments in self-control and inhibition often accompanied by decreased or ineffective prefrontal cortical recruitment.^4, 5^ Moreover, the fronto-striatal systems affected by cocaine use^6^ and compromised in chronic drug users overlap considerably with those mediating cognitive control and executive functioning in healthy individuals.^3^

A commonly held view suggests that along with ongoing cocaine use, its reinforcing nature leads to eventual escalation of intake.^7^ Nevertheless, intact or even enhanced control may enable some individuals to regulate cocaine use, enabling them to curtail such escalation. Cognitive control could thus be important in allowing those individuals who engage in prolonged occasional use to escape transition to abuse and dependence. These recreational cocaine users fail to satisfy the criteria for cocaine dependence or abuse and do not seek treatment. There is indeed some evidence that cognitive control may not be impaired in some recreational users, especially socially integrated consumers who use the drug infrequently, in small amounts, and typically in social contexts.^8^ These individuals appear to differ substantially from cocaine-dependent users not only in their tightly regulated patterns of use^9^ but also in their apparent absence of psychological or physiological signs associated with cocaine abuse.^10^ Their anecdotal ability to prioritize work or school above drug-taking suggests they may have enhanced capacities for self-control and future planning, even compared with the general population.

Another likely factor in recreational users is their apparent lack of vulnerability factors characterizing stimulant-dependent individuals such as early-life trauma, increased impulsivity and compulsivity and early drug exposure.^8^ This is consistent with evidence that patterns of use and the effects of stimulants may interact extensively with pre-existing individual genetic and psychosocial characteristics.^11^ Therefore, individual traits and early environment together with intact, or even enhanced, cognitive control may curb the transition to dependence. By investigating recreational cocaine users, better identification of those likely to transition to dependence can be achieved, allowing for more effective risk markers and interventions. Examining those who do not transition can also elucidate the mechanisms of resilience and compensatory processes to the aversive effects of cocaine. Moreover, such research could offer evidence regarding the cognitive effects of protracted cocaine in humans, as these remain controversial.^12, 13^

One key aspect of cognitive control is response inhibition, or the ability to intentionally stop planned or ongoing actions when they are no longer appropriate. Difficulties in stopping are believed to contribute to impulsivity, a construct tightly linked with maladaptive cocaine use.^14^ The ability to stop and not escalate stimulant drug seeking or taking, despite their positive reinforcing actions, could typify recreational users. Response inhibition constitutes a particularly useful assay of cognitive control and is mediated by defined fronto-striatal circuits with prefrontal cortical involvement specifically including the pre-supplementary motor area (SMA) in the dorsomedial prefrontal cortex (dmPFC), and the ventrolateral PFC (vlPFC).^15, 16^ Previous evidence has indicated impaired response inhibition in chronic stimulant drug users, accompanied by reduced vlPFC recruitment.^17, 18^ The unaffected biological siblings of these stimulant drug users also exhibited difficulties in stopping prepotent responses, but rather than showing concomitantly reduced vlPFC activity, this difficulty was accompanied by increased dmPFC recruitment suggestive of compensatory mechanisms for pre-existing vulnerability factors.^18^

Reports on response inhibition in non-dependent stimulant drug users have been mixed with one initial small study showing inhibitory impairment^19^ but subsequent studies indicating no performance difficulties, and only negligible functional activation alterations compared with healthy controls being occasionally noted.^20, 21^ Such results are reminiscent of the mixed results of studies investigating other prefrontal cortex-mediated top-down functions such as working memory, selective attention and planning.^22, 23, 24, 25^ Some degree of impairment may be consistent with the notion that occasional users are at different points along a trajectory towards dependence. Accordingly, there is evidence that intense recreational cocaine users show high levels of impulsivity^21^ and lower cognitive performance,^26^ though less severe compared with dependent cocaine users. The mixed findings also highlight the challenge of characterizing this population given the current lack of clear criteria or clinical guidelines for classifying recreational and occasional use of cocaine.^27^

The present study examined performance during a stop-signal task to assess response inhibition and its neural correlates using functional magnetic resonance imaging in suitably large numbers of recreational cocaine users and healthy controls. The stop-signal task requires cancellation of a planned response in a neutral setting, despite a strong tendency to carry it out to completion. We used the same paradigm as that in a previous study of chronic stimulant users and their unaffected biological siblings,^18^ hypothesizing that brain functioning might provide a more sensitive measure to any possible aberrations in response inhibition.

The recreational users were a relatively homogeneous group with no family history of abuse, no psychiatric comorbidities, who reported controlled recreational use with no interference in daily functioning, although they had been using cocaine for at least 2 years. Data from this group have already suggested a markedly different neurobiological phenotype from that found in stimulant-dependent users, with unimpaired attentional bias to cocaine-related stimuli accompanied by reduced prefrontal activation compared with stimulant-dependent and control individuals.^27^ Moreover, divergent prefrontal structural abnormalities between recreational and stimulant-dependent users have been reported with increased rather than decreased orbitofrontal gray matter in the former, (though some commonalities in brain abnormalities were also noted).^8^ Thus, we hypothesized that response inhibition may be a factor contributing to apparent resilience in recreational users. We investigated whether or not they would demonstrate difficulties suppressing actions and whether this would be accompanied by aberrations in key prefrontal regions, including the dmPFC and vlPFC.

## Materials and methods

### Participants

Recruitment and screening procedures have been described in detail elsewhere.^8, 27^ All participants were aged 18–55, with no history or current psychiatric, neurological or neurodevelopmental disorder or traumatic brain injury. Inclusion criteria included no family history of substance dependence, with the exception of nicotine, and no current psychotropic medication. All participants were evaluated using the Structured Clinical Interview for DSM-IV^28^ augmented with a semi-structured interview to ascertain history of drug use, mental and physical health. Recreational drug users used cocaine for at least 2 years without experiencing physiological or psychological symptoms of dependence as described in the DSM-IV and did not use stimulant drugs for medical reasons. Recreational users had never developed DSM-IV criteria for substance dependence, having used cocaine in relatively small amounts in powdered forms in social settings infrequently (see Table 1). Their occasional use did not interfere with work, school, family or social obligations and they never considered seeking any treatment (see Supplementary Information for further details). Twenty-seven recreational users were recruited from the community by local advertisements, though three were excluded (see Supplementary Information). A sample of 32 controls was matched in age, gender and education.

Drug urinalysis was collected on testing day and results were negative for all the participants. Verbal intelligence quotient (IQ) was assessed by the National Adult Reading Test,^29^ depressive mood by the Beck Depression Inventory-II,^30^ impulsivity by the Barratt Impulsiveness Scale-11 (ref. 31) and sensation-seeking traits by the Sensation-Seeking Scale-Form V.^32^ Alcohol use was quantified by the Alcohol Use Identification Test^33^ and obsessive-compulsive tendencies by the Padua Inventory-Revised.^34^ In the recreational users, compulsive drug-taking was further assessed with the Obsessive-Compulsive Drug Use Score (OCDUS).^35^ The study was approved by the Cambridge Research Ethics Committee (REC08/H0308/310; principal investigator, KDE), and before participation, volunteers provided written informed consent.

### Stop-signal task

The task was identical to that reported in a previous study.^18^ Participants viewed the task stimuli via a mirror as they lay in the scanner. On go trials, participants pressed left and right buttons in response to go stimuli (left and right pointing white arrows, 1000 ms). On stop trials, the go stimulus was followed by a visual stop signal (orange arrow pointing upwards, 300 ms) and participants had to withhold responding. There were 48 stop trials and 240 go trials, presented intermixed and counterbalanced with left and right, in a single block with three to seven go trials between stop trials. The delay between go and stop stimuli, initially set to 250 ms, was adjusted individually by a tracking algorithm in 50-ms steps to allow 50% successful stopping.^36^ If a response was recorded before stop-signal onset, it did not appear and the trial was repeated (<1% of trials). Intertrial intervals were randomly jittered between 700 and 1100 ms.

### Scanning acquisition

Whole-brain echo planer images were collected in one run on a Siemens TIM Trio 3-Tesla scanner with the following parameters: repetition time=2000 ms; echo time=30 ms; flip angle=78º 32 slices with slice thickness 3 mm, 0.75 mm gap; matrix=64 × 64; field of view=192 × 192 mm yielding 3 × 3 mm in-plane resolution, and the number of volumes ranging from 274 to 299. T1-weighted scans were acquired for registration (176 slices of 1 mm thickness, repetition time=2300 ms; echo time=2.98 ms, inversion time=900 ms, flip angle=9°, field of view=240 × 256 mm).

### Data analysis

Behavioral analyses compared recreational users and controls in mean go reaction time (RT) and stop-signal RT (SSRT). SSRT was estimated by subtracting mean stop-signal delay from correct go RT in accordance with the race model.^37^ In addition, percent unsuccessful stopping was computed and unsuccessful stop RT was compared with go RT. Exclusion criteria to ensure the race model was adopted^18^ resulted in the exclusion of three recreational users. Behavioral and demographic data were analyzed using chi-squared and *t*-tests and significant group differences are followed by Cohen d's effect-size.

Imaging data were processed and analyzed using Statistical Parametric Mapping 8 (http://www.fil.ion.ucl.ac.uk/spm/). The first five volumes were discarded due to T1-equilibrium effects. Images were realigned and mean echo planer image was co-registered to the T1-weighted image, which was segmented and warped to the Montreal Neurological Institute template using New Segment and the deformations applied to the echo planer image volumes which were resampled to 2 × 2 × 2 mm. Finally, images were smoothed with a Gaussian kernel of 8-mm full width half maximum.

First-level analyses were performed using the general linear model in Statistical Parametric Mapping 8. Individual design matrixes modeled successful stop, unsuccessful stop and erroneous go trials by convolving onset times with a canonical hemodynamic response function with temporal and dispersion derivatives. Correct go trials occurred frequently, comprising the general linear model baseline. First-level contrasts were computed for stop relative to baseline, and failed versus successful stops. Anatomical regions of interest examining group differences associated with stopping included the right anterior insula/frontal operculum (comprising pars opercularis, pars triangularis and anterior insula with y>0) and right and left pre-SMA (y>0) and anterior cingulate cortex (ACC) from the automated anatomical labeling atlas (see Supplementary Figure S1).^38^ All results reported were significant at *P*<0.05 corrected for family-wise error with small volume correction, and peak voxels in Montreal Neurological Institute coordinates. Where significant between-group differences were found, eigenvariates were extracted from 8-mm spheres surrounding peak coordinates for each individual, and were correlated with task performance using Pearson correlation coefficients. Results were further examined while covarying for tobacco and alcohol consumption. To investigate the broader set of regions associated with overriding prepotent responses, group differences in mean activation were computed for an independent search area region of interest constructed from all 8-mm spheres surrounding the coordinates derived from a meta-analysis^39^ using MarsBar^40^ (see also Supplementary Information).

## Results

### Demographic and clinical measures

The groups did not differ on gender distribution, age, education and verbal IQ (see Table 1). Recreational users scored higher than controls on self-reported measures of sensation-seeking and alcohol use and marginally higher on impulsivity. They also scored somewhat lower on obsessive-compulsive and higher on depression severity measures compared with controls, though scores in both groups were very low. Although recreational cocaine use duration was ~8 years, their OCDUS indicated very low compulsive use, consistent with their low obsessive-compulsive severity.

### Behavioral measures

As seen in Table 2, there were no significant group differences on any task performance indices, with SSRTs being nearly identical. In keeping with the assumptions of the race model, both recreational users (*t*(23)=5.08, *P*<0.01) and controls (*t*(31)=2.93, *P*<0.01) had faster unsuccessful stop than go latencies.

### Neuroimaging

**Figure 1:**
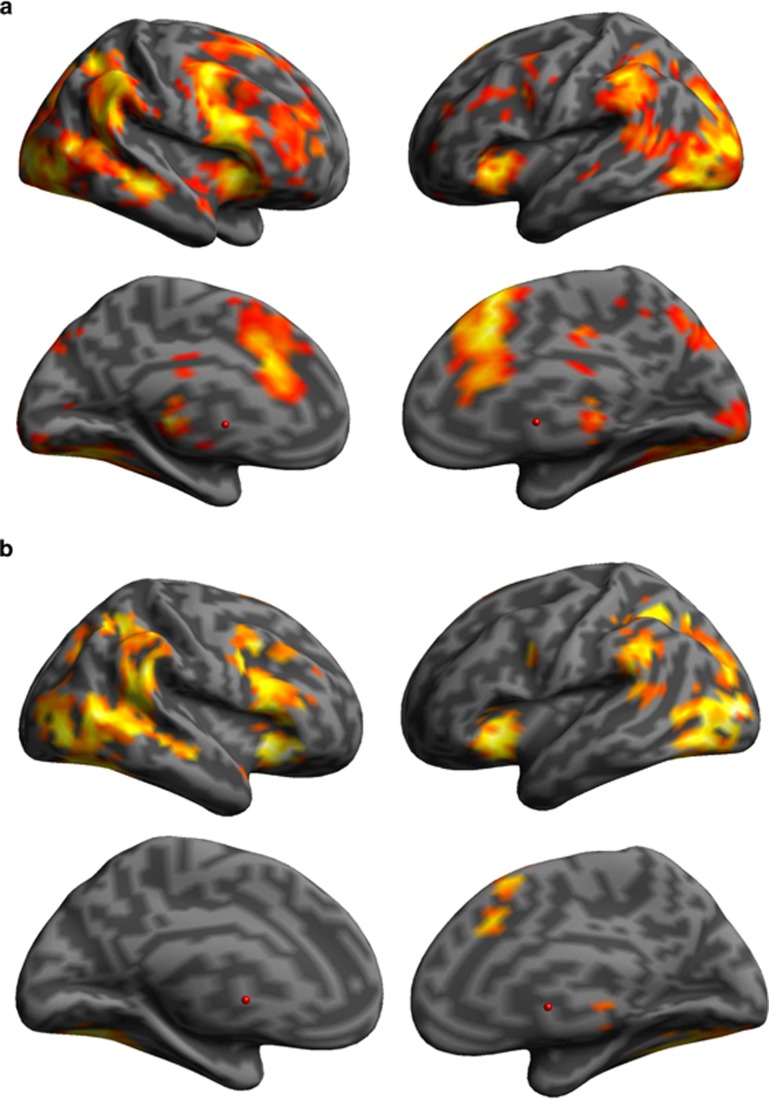
Significant brain activation maps associated with stopping in each group separately. (**a**) Denotes lateral and medial views of recreational cocaine users. (**b**) Denotes lateral and medial views of healthy volunteers. *P*<0.001 uncorrected, for illustration purposes only.

Each group demonstrated activation in regions commonly observed in this task for stopping in whole brain analyses corrected for family-wise error, *P*<0.05. These included the vlPFC, encompassing the anterior insula and IFG predominantly on the right, in addition to the dmPFC, superior inferior parietal cortex and occipital cortex bilaterally (see Figure 1). Visual inspection suggested greater and more extensive activation in recreational users compared with controls during stopping. In accordance with this conclusion, the anatomical region of interest analyses indicated overactivation in recreational users compared with the controls in stopping versus going in the right pre-SMA (*P*=0.048, [10,20,46], cluster extent (*K*_E_)=8, *Z*=3.23). In addition, there was increased activation in the right ACC (*P*=0.028, [−2,26,30], *K*_E_=24, *Z*=3.58) and left ACC (*P*=0.047, [4,24,18], *K*_E_=14, *Z*=3.58) for stopping. These results remained when covarying for alcohol and tobacco consumption (see Supplementary Information for additional details). There was no significant group difference in the vlPFC region of interest. In addition, no group differences were noted in failed versus successful stopping (see Supplementary Information for additional details). Recreational drug users showed increased activation in the response override search area (*t*(54)=1.86, Contrast Value 0.96, *P*=0.034), though both groups showed significant activation compared with the go baseline (*t*(31)=4.43, *P*<0.001 and *t*(23)=6.45, *P*<0.001 for controls and recreational users, respectively). SSRT did not correlate significantly with any functional activation or group characteristic. In sum, recreational users showed increased activation not only in the pre-SMA but also more general widespread activation in areas associated with suppressing prepotent responses compared with controls.

## Discussion

This study examined response inhibition, as gauged by the stop-signal task and its neural correlates using functional magnetic resonance imaging, in well-characterized recreational cocaine users and matched controls. The behavioral performance in the recreational group was on par with that of the healthy controls, with no difference on any task measure, including response execution and SSRT. At the same time, within the neural circuitry normally activated by stop-signal and response override tasks, recreational users showed significantly increased activation, including the dmPFC and ACC.

These findings reinforce the notion of a neurobehavioral phenotype in the recreational cocaine users that is distinct from that shown in chronic stimulant-dependent users, who demonstrate performance difficulties in response inhibition along with reduced vlPFC recruitment.^17, 18^ Similarly, recreational users did not show evidence for altered error processing as previously reported in stimulant-dependent individuals.^18, 41, 42^ Previously, it was shown that recreational users also exhibit increases rather than the decreases in orbitofrontal gray matter that characterize stimulant-dependent individuals.^8^ In contrast to stimulant-dependent individuals, recreational users also did not exhibit attentional bias to cocaine-related stimuli in conjunction with reduced prefrontal and orbitofrontal activations.^27^ Thus, prefrontal cortical and control processes do not appear to be impaired in the same way in these two cocaine-using groups, with several apparently opposed patterns of abnormalities evident, compatible with their divergent usage patterns. This conclusion dovetails with the findings that the underlying substrates of response inhibition such as the dmPFC and vlPFC show no overlapping abnormalities in structure between these two groups, though abnormally increased gray matter volume in the parahippocampus gyrus has been reported for both groups.^8^

Increased recruitment of areas key to response inhibition in the presence of equivalent performance suggests compensatory or protective neural mechanisms in the recreational users. Moreover, it is strongly reminiscent of (though not identical to) the findings reported for the unaffected siblings of stimulant-dependent individuals.^18^ In both cases, adequate performance was accompanied by increased dmPFC but no abnormalities in vlPFC functional activation. Present results also add to the similarity between the two groups in increased cerebellar gray matter.^8^ These similarities between the non-dependent siblings and recreational drug users and the differences between these two groups on one hand and the drug-dependent individuals on the other, strengthen the notion that unlike measures of brain structure, functional activations during response inhibition may not be a suitable endophenotype for drug dependence,^18^ although they could provide a marker for the capacity for functional compensation. The present functional magnetic resonance imaging data alone do not distinguish between results indicating inefficient neural recruitment, as opposed to marking the resilience that allows individuals to avoid cocaine dependence (akin to the unaffected siblings avoiding cocaine altogether). However, PFC hyperactivation reminiscent of the present findings has been reported during stop-signal task performance in obsessive compulsive disorder patients, their unaffected first-degree siblings and in adolescents reporting limited use of illicit substances.^43, 44^ Taken together, such hyperactivation in the absence of performance difficulties may be markers of general compensatory mechanisms. Thus, some level of vulnerability or compromise to the system, from pre-existing susceptibility or from limited drug exposure, can be gauged by the apparently greater functional recruitment. However, such compensation may only be possible to a certain extent, as with increasing usage or with greater vulnerability, it becomes no longer viable resulting in reduced neural recruitment and disrupted performance. An alternative account might suggest that behavioral measures are less sensitive than brain activations and that current findings might point to reduced neural recruitment in controls. This would be congruent with the notion of resilience in the present recreational users who may have exceptional capacities for cognitive control. Further, the chronic, though limited, use of cocaine in this group could have eroded their potentially superior performance. However, considering increased recruitment of key prefrontal regions in the absence of behavioral differences in the unaffected siblings and other groups, we believe the former interpretation of compensatory recruitment to be more parsimonious. Future functional studies examining recreational cocaine users longitudinally or their first-degree non-using relatives may elucidate the issue. Increased activation in the response override search area is suggestive of broad compensatory recruitment. However, the contrast between stop and go engages not only response inhibition but also attentional orienting as the stop signal occurs infrequently,^39^ and both processes have been associated with the inferior frontal cortex.^39, 45^ Nonetheless, the pre-SMA is routinely linked to action suppression *per se* in the stop signal task^46^ and more broadly to action control^47^ during response-inhibition tasks, suggesting some regional specificity for compensatory recruitment. The recreational users were well characterized, exhibiting no current or past psychiatric disorders and without a family history of dependence. Further, the two groups were matched for childhood trauma.^8^ Most investigations of occasional cocaine users likely include variable mixtures of stable recreational users and individuals on a trajectory to dependence, leading to difficulties in integrating findings across studies. Some recreational user studies have included primarily males or individuals who may have previous psychiatric comorbidities such as attention-deficit hyperactivity disorder or alcohol abuse, which increase the likelihood of transition to dependence.^19, 21^ Moreover, male recreational users may be particularly prone to poorer cognitive performance.^48^ The average age of participants is considerably lower in some studies as is duration of usage (for example, minimum of 6 versus 24 months) allowing a substantial proportion of individuals who may subsequently escalate.^20^ Finally, our sample of recreational users included individuals with higher levels of education, IQ and disposable income than those reported for stimulant users,^8^ in other studies the two groups were more comparable with high levels of craving and impulsivity. Indeed, a proportion of recreational cocaine users in a longitudinal study subsequently increased their usage.^13, 21^ Nevertheless, in accordance with present findings, two previous studies reported no differences in performance on the stop-signal task, reinforcing the conclusion that cocaine use in humans does not necessarily lead to inhibition performance deficits,^20, 21^ although detrimental effects on other domains such as working memory may be apparent.^26^ At the same time, a study using a mixed group of young cocaine and prescription stimulant users reported weaker recruitment during stopping compared with controls of parietal and cingulate regions, at odds with the present findings.^20^ Should increased activation in parts of the response override network be indicative of preliminary compensatory recruitment as suggested above, it is predicted that longitudinal studies would reveal initial overactivation to be followed by hypoactivation and performance difficulties. Such longitudinal studies of recreational users are particularly important as they can address the extent to which compensatory strategies may be successful and provide insight into how such strategies fail. Possibilities include the accumulation of the drugs consumed or interference from environmental stressors that could interfere with the cognitive resources necessary for compensatory strategies. Better insight into such issues would have important implications for preventative strategies. In any case, we argue that finer distinctions should be adopted to better characterize what are potentially non-overlapping populations of recreational users. It would also be of use to compare performance and neural activation in recreational users to that of abstinent users as inhibitory control may contribute in a similar fashion to relapse avoidance. Evidence regarding performance in abstinent users typically encompasses a range of abstinence durations, and is limited in part due to the challenges of conducting such studies. Response inhibition appears impaired in some studies^49^ but not others.^42^ Though hyperactivation of some PFC regions was initially reported in one small study,^50^ this was not replicated subsequently,^51, 52^ while ACC hypoactivation akin to drug users has also been noted.^42^ Thus, on the whole, it remains to be determined whether successful abstainers can recruit additional prefrontal resources as present recreational users, although such mechanisms may be exploited more effectively with the adoption of training regimes to bolster response inhibition. As a psychological construct, response inhibition is believed to contribute to cognitive control and executive functioning.^53^ As such, it may interact in complex ways with overlapping factors implicated in promotion of stimulant dependence. Familial vulnerability has a key role as does age of initial use, with greater likelihood of transition to dependence with earlier exposure.^54^ This account is consistent with response inhibition and its mediating neural structures still undergoing maturation until early adulthood.^55, 56^ Disruptions of behavioral inhibition and top-down control may be a key mechanism by which additional factors such as the socio-demographic environment and intelligence contribute to the likelihood of becoming dependent.^57, 58^ Hence, response inhibition and controlled intake may mutually promote one another, reducing the likelihood of transitioning to dependency. Sensation-seeking, while being a strong predictor for drug use,^8, 59^ appears to be orthogonal both to response inhibition and to the likelihood of escalation to dependence. This is because increased sensation-seeking characterizes the recreational users in addition to the stimulant-dependent individuals but not their unaffected siblings. As such it would be useful to investigate reward processes more closely in recreational users.^60^

Study limitations include the cross-sectional design and the lack of clinical guidelines defining controlled or recreational cocaine use. Nevertheless, the two groups were well-matched for age, gender, childhood adversity, education and intelligence levels. The task did not model go trials separately and so was probably insensitive to striatal involvement in response control, although its use enabled opportunities to integrate both present and previous results.^18, 44^ Although we utilized urine analysis and comprehensive psychiatric diagnostics, cocaine use rested largely on self-report. Future studies should quantify longer-term cocaine use with objective measures such as hair toxicology. We did not seek estimates for the amount of cocaine used as this may be confounded with purity in addition to the usual shortcomings of relying on retrospective reporting. The present findings also do not preclude significant difficulties in other domains of top-down control in the recreational cocaine users such as working memory or other cognitive domains mediated by neural structures showing similar abnormalities in recreational users and stimulant-dependent individuals such as the hippocampus.^8^

In summary, response inhibition performance of recreational cocaine users who had been using cocaine for at least 2 years with no family history of abuse or psychiatric comorbidities was on par with matched controls. Nevertheless, intact stopping in the recreational users was accompanied by overactivation of the dmPFC, reminiscent of the unaffected siblings of stimulant-dependent individuals. A parsimonious account favors the increased activity as indicative of compensatory recruitment of key response-override brain regions. The results suggest that fostering cognitive control in occasional cocaine users may enable such individuals to delay or avoid possible transition to dependence.

## Figures and Tables

**Table 1:** Demographic information and group differences for recreational stimulant users and healthy control subjects

*Characteristic*	*Recreational users*		*Control subjects*		t/χ^*2*^	P
	*Mean*	*s.d.*	*Mean*	*s.d.*		
Male: female	12:12		18:14		0.22	0.64
Age (years)	28.54	6.78	30.91	8.14	1.15	0.25
Education (years)	13.29	1.78	13.03	1.99	0.51	0.61
Verbal IQ (NART)	116.32	5.25	113.58	8.12	1.39	0.17
Impulsivity (BIS-11)	62.92	10.68	58.44	7.25	1.87	0.07
Sensation seeking	23.04	5.13	18.28	5.91	3.15	<0.01
Compulsivity (Padua Inventory)	4.33	2.82	7.72	7.45	2.11	0.04
Depression (BDI-II)	4.04	4.43	2.47	2.18	1.75	0.09
Mean number of cigarettes	5.17	5.78	4.25	6.59	0.34	0.74
Alcohol use (AUDIT)	5.79	1.56	3.00	2.31	5.10	<0.01
Compulsivity (OCDUS)	1.25	1.67				
Duration of stimulant use (years)	8.08	6.18				
Age of stimulant use onset (years)	20.42	3.39				

Abbreviations: AUDIT, Alcohol Use Disorders Identification Test, cut-off score for alcohol abuse >8; BDI-II, Beck Depression Inventory–II; BIS-11, Barratt Impulsivity Scale Version 11; IQ, intelligence quotient; NART, National Adult Reading Test; OCDUS, stimulant-related Obsessive-Compulsive Drug Use Scale.

**Table 2:** Stop-signal task performance measures

*Task measure*	*Recreational*		*Control subjects*		t	P
	*Mean*	*s.d.*	*Mean*	*s.d.*		
Go RT (ms)	386.53	46.30	410.91	76.17	1.38	0.17
SSRT (ms)	198.36	36.92	198.38	59.12	0.01	0.99
Percent errors (on go trials)	3.71	3.19	3.17	2.43	0.72	0.47
Percent unsuccessful stopping	50.13	1.70	49.11	2.18	1.91	0.06
Go s.d. (ms)	90.81	24.69	91.54	34.71	0.09	0.93
Slowing following an unsuccessful stop (ms)	22.82	36.21	30.04	54.82	1.13	0.26

Abbreviations: RT, reaction time (in ms); SSRT, stop signal reaction time (in ms).

Go s.d. denotes the individual standard deviation of go RT.

## Notes

TWR consults for Cambridge Cognition, Lilly, Lundbeck and GlaxoSmithKline and also holds research grants from those companies. ETB is employed part-time by GlaxoSmithKline and part-time by the University of Cambridge. The remaining authors declare no conflict of interest.
