# Supplementary material for: Take it or leave it: prefrontal control in recreational cocaine users
Source: Transl Psychiatry. 2015 Jun 16;5(6):e582–. doi: 10.1038/tp.2015.80 (PMC4490290; doi:10.1038/tp.2015.80)

**Supplementary Materials**

*Additional details about the recreational cocaine users*

The recreational users almost all (96%) reported a lifetime history of sporadic, controlled experimentation with illicit drugs other than cocaine, including hallucinogens (50%), amphetamine (58%), ecstasy (87%) and cannabis (100%), but never fulfilled the DSM-IV-TR criteria of substance dependence. None reported any use of opiates, steroids or Gamma Hydroxybutyrate. This pattern of experimentation was consistent with their overall selective and controlled experimentation and usage in social settings with friends and their high sensation seeking (see also low DAST scores in Table 1).

Adult ADHD comorbidity was not ascertained explicitly, but it is highly unlikely that any individuals qualified for ADHD as: a) none reported a childhood history or diagnosis, b) none reported being prescribed ADHD medications, c) none reported using stimulant drugs to ‘calm down’, and d) present results did not find evidence for common findings in ADHD such as elevated impulsivity, increased depression or impaired performance on stop-signal task measures ^1^. Moreover, individuals with ADHD are typically characterized by underactivation of prefrontal areas accompanied by overactivation of more posterior regions, which is considerably different from the present profile ^2^.

*Stop signal task and analysis*

The key dependent measures in the stop signal task are go reaction time (RT), which provides a measure of motor initiation, and stop signal RT (SSRT), which provides a measure of response inhibition. When using the tracking algorithm, convergence around 50% success rate on stop trials together with the race model allows for estimation of SSRT by subtracting mean stop signal delay from go RT. Secondary measures include individual go standard deviation (SD) as an attentional measure and slowing following unsuccessful stop as a measure of error monitoring. Criteria adopted to uphold the race-model were identical to those previously used in our group ^3^, These included: a) 0.4 to 0.6 successful inhibition, b) a difference between unsuccessful stop and go RT below 1 SD of the sample, c) less than 30 trials where a response occurred prior to a planned stop-signal (the stop-signal was present in 15.1% to 16.6% of trials).

*Additional fMRI analyses*

We investigated whether recreational users differed from controls in the amount of head motion in the scanner. There was no significant difference in frame displacement (dvars, http://fsl.fmrib.ox.ac.uk/) between the two groups (t(1,53)=1.3, p=0.18).

Figure S1 conveys the anatomical ROIs used in the main text. An independent region of interest to examine frontal activation associated with response inhibition was derived based on coordinates from a meta-analysis ^4^. The activation likelihood estimation analyses comprised motor inhibition tasks specifically where the motor tasks were prepared or initiated prior to stimulus onset (response override tasks), indicating the need to suppress a prepotent response. For each of the coordinates reported in the analysis (based on Table 5 in the original publication, and presented in Table S1) an 8-mm sphere was created in MarsBar ^5^ and the resulting regions of interest were combined to create the search area.

The recreational users had slightly higher verbal intelligence and levels of self-reported impulsivity (see Table 1). Analyses of stop signal performance covarying for these two factors revealed the same results reported in the main text. Similarly, analyses of eigenvariate values when covarying for the BIS (Barratt Impulsiveness Scale) and verbal IQ again did not alter the results reported in the main text, with increased activations associated with stopping in recreational users compared to controls in the right pre-SMA (F(1,53)=10.70, p=0.002), and right and left ACC (F(1,53)=8.35, p=0.006 and F(1,53)=9.41, p=0.004, respectively).

Comparing unsuccessful to successful stops in the ACC ROI revealed significant activation across all participants when correcting for family wise error (p=0.005, [0, 34,8], K_E_=70, Z=4.28, and p=0.025, [2,54,12], K_E_=35, Z=3.87). However, there was no evidence for significant differences between the two groups, even at a threshold of p<0.001 uncorrected. Similarly, whole brain analyses at the threshold of p<0.001 uncorrected did not detect any group differences. Along with the absence of group differences in behavioral indices relating to monitoring following unsuccessful stops (see Table 1), present data do not support abnormal error monitoring in the recreational cocaine.

To further explore potential differences between the groups, we conducted whole brain analyses at a more liberal threshold of p<0.001 uncorrected. The results, reported in Table S2, indicated scattered foci of increased activation associated with stopping in the recreational users compared to the control participants. These primarily centered on the prefrontal cortex (including the pre-SMA and anterior cingulate as reported in the main text). Additional foci were noted in the occipital cortex and fusiform gyrus, likely stemming from the visual nature of the stop signal.

*Tobacco and Alcohol*

As noted in Table 1 in the main text, the recreational users reported higher alcohol consumption and controls as assessed by the AUDIT (Alcohol Use Disorders Identification Test), though below the cutoff for abuse, and did not differ in daily cigarette use. Analyses of eigenvariate values when covarying for AUDIT scores indicated that recreational users had increased activations associated with stopping compared to controls in the right pre-SMA (F(1,53)=5.02, p=0.029). Similarly, increased activation in the right and left ACC remained significant (F(1,53)=4.47, p=0.039 and F(1,53)=6.28, p=0.015, respectively).

*Supplementary references*

1. Lijffijt M, Kenemans JL, Verbaten MN, van Engeland H (2005). A meta-analytic review of stopping performance in attention-deficit/hyperactivity disorder: deficient inhibitory motor control? J Abnorm Psychol 114(2): 216-222. Hart H, Radua J, Nakao T, Mataix-Cols D, Rubia K (2013). Meta-analysis of functional magnetic resonance imaging studies of inhibition and attention in attention-deficit/hyperactivity disorder: exploring task-specific, stimulant medication, and age effects. JAMA Psychiatry 70(2): 185-198.
2. Morein-Zamir S, Simon Jones P, Bullmore ET, Robbins TW, Ersche KD (2013). Prefrontal hypoactivity associated with impaired inhibition in stimulant-dependent individuals but evidence for hyperactivation in their unaffected siblings. Neuropsychopharmacology. 38:1945-1953.
3. Levy BJ, Wagner AD (2011). Cognitive control and right ventrolateral prefrontal cortex: reflexive reorienting, motor inhibition, and action updating. Annals of the New York Academy of Sciences. 1224:40-62.
4. Brett M, Anton J, Valabregue R, Poline JB (2002). Region of interest analysis using an SPM toolbox. Abstract presented at the 8th International Conference on Functional Mapping of the Human Brain. June 2–6, Sendai, Japan.

**Table S1**. Coordinates from frontal regions that comprised the search area for response inhibition tasks requiring suppression of a prepotent response.

| Anatomical label | Hemisphere | x | y | z |
| --- | --- | --- | --- | --- |
| Anterior insula | R | 38 | 20 | -2 |
|  | L | -42 | 16 | -6 |
| Inferior frontal gyrus | R | 48 | 16 | 18 |
| Pre-supplementary motor area | R | 14 | 16 | 58 |
| Middle Frontal gyrus | R | 34 | 42 | 28 |
|  | L | -36 | 38 | 24 |
| Anterior cingulate | R | 6 | 22 | 36 |
| Middle frontal gyrus | L | -38 | 54 | 18 |
| Dorsal premotor cortex | R | 30 | 0 | 44 |
|  | L | -26 | -4 | 52 |

**Table S2.** Group differences in activations associated with stopping at whole brain p<0.001 uncorrected extent threshold 10 voxels.

| Hemisphere | Z-score | Peak coordinates MNI (mm) | | | Cluster size (voxel) | Brain Region |
| --- | --- | --- | --- | --- | --- | --- |
|  |  | x | y | z |  |  |
| R | 3.36 | 4 | 24 | 18 | 27 | Anterior Cingulate |
| L | 3.58 | -2 | 26 | 30 | 42 |  |
| R | 3.23 | 10 | 20 | 46 | 14 | Pre-SMA |
| R | 3.35 | 12 | -18 | 34 | 12 | Middle Cingulate |
| R | 3.54 | 10 | 54 | 26 | 21 | Superior frontal gyrus |
| L | 3.51 | -10 | 58 | 14 | 44 |  |
| R | 3.34 | 16 | 46 | 24 | 21 | Superior medial gyrus |
| L | 3.27 | -32 | 54 | 2 | 13 | Superior middle gyrus |
| L | 3.47 | -32 | -8 | 34 | 10 | Precentral gyrus |
| R | 3.44 | 16 | -38 | 10 | 13 | Hippocampus |
| R | 3.32 | 34 | -74 | -4 | 11 | Inferior Occipital |
| R | 3.37 | 30 | -60 | 20 | 20 | Occipital |
| L | 3.73 | -30 | -68 | -4 | 32 | Fusiform |
| L | 3.50 | -32 | -50 | -4 | 13 | Fusiform |

Figure S1. The anatomical region of interest volumes (right and left pre-supplementary motor area and anterior cingulate and right inferior frontal cortex comprising of the inferor frontal gyrus and anterior insula).


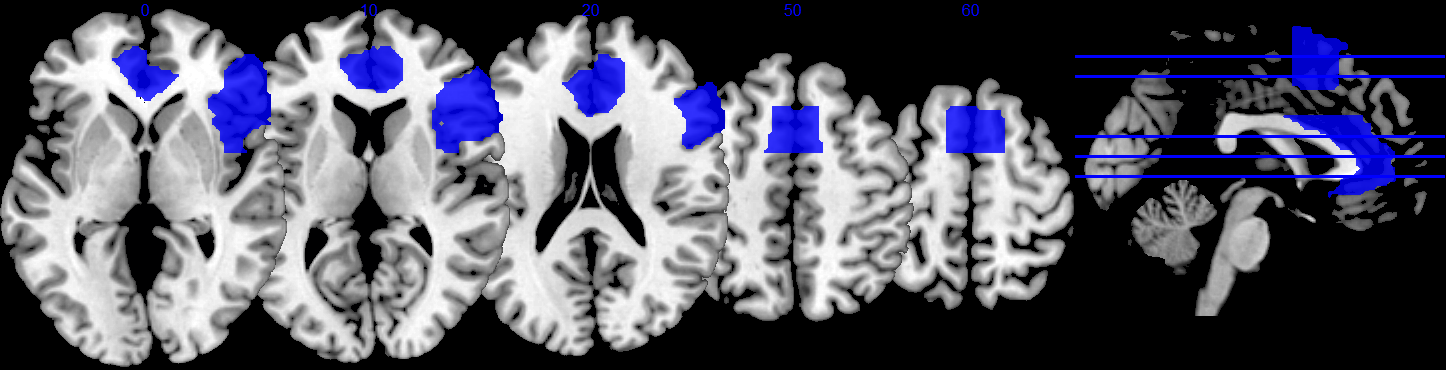

Supplement: Supplementary Information [file tp201580x1.doc]
